# Expression Profile of Genes as Indicators of Developmental Competence and Quality of *In Vitro* Fertilization and Somatic Cell Nuclear Transfer Bovine Embryos

**DOI:** 10.1371/journal.pone.0108139

**Published:** 2014-09-30

**Authors:** Maria Jesús Cánepa, Nicolás Matías Ortega, Melisa Carolina Monteleone, Nicolas Mucci, German Gustavo Kaiser, Marcela Brocco, Adrián Mutto

**Affiliations:** 1 Laboratorio de Biotecnologías Reproductivas y Mejoramiento Genético Animal, Instituto de Investigaciones Biotecnológicas, Universidad Nacional de San Martín, General San Martín, Prov. de Buenos Aires, Argentina; 2 Laboratorio de Biotecnología de la Reproducción, Instituto Nacional de Tecnología Agropecuaria, INTA Balcarce, Prov. de Buenos Aires, Argentina; USA, United States of America

## Abstract

Reproductive biotechnologies such as *in vitro* fertilization (IVF) and somatic cell nuclear transfer (SCNT) enable improved reproductive efficiency of animals. However, the birth rate of *in vitro*-derived embryos still lags behind that of their *in vivo* counterparts. Thus, it is critical to develop an accurate evaluation and prediction system of embryo competence, both for commercial purposes and for scientific research. Previous works have demonstrated that *in vitro* culture systems induce alterations in the relative abundance (RA) of diverse transcripts and thus compromise embryo quality. The aim of this work was to analyze the RA of a set of genes involved in cellular stress (heat shock protein 70-kDa, HSP70), endoplasmic reticulum (ER) stress (immunoglobulin heavy chain binding protein, Bip; proteasome subunit β5, PSMB5) and apoptosis (BCL-2 associated X protein, Bax; cysteine aspartate protease-3, Caspase-3) in bovine blastocysts produced by IVF or SCNT and compare it with that of their *in vivo* counterparts. Poly (A) ^+^ mRNA was isolated from three pools of 10 blastocysts per treatment and analyzed by real-time RT-PCR. The RA of three of the stress indicators analyzed (Bax, PSMB5 and Bip) was significantly increased in SCNT embryos as compared with that of *in vivo*-derived blastocysts. No significant differences were found in the RA of HSP70 and Caspase-3 gene transcripts. This study could potentially complement morphological analyses in the development of an effective and accurate technique for the diagnosis of embryo quality, ultimately aiding to improve the efficiency of assisted reproductive techniques (ART).

## Introduction

Recent studies regarding the outcome of *in vitro* fertilization (IVF) and somatic cell nuclear transfer (SCNT) have demonstrated that embryonic developmental competence can be severely compromised without apparent correlation with morphological changes. Good quality embryos classified according to morphological criteria may have different developmental capacities, with only a certain percentage of these embryos being capable of establishing pregnancy after transfer into recipients [1]. In addition, developmental competence of *in vitro*-produced embryos following transfer is significantly lower than that of those produced *in vivo* [2]. In conclusion, qualitative or morphological evaluation alone is not sufficient in providing a precise and efficient estimation of embryo quality and embryonic developmental potential [3].

In modern cattle production, there is vast information demonstrating that micromanipulation and embryo culture conditions can have a dramatic effect on gene expression patterns, which can be disadvantageous to the quality of the resulting blastocyst [4]–[14].

Several studies have proven the broad applicability of real-time reverse transcription polymerase chain reaction (qRT-PCR) for differential gene expression studies. Researchers have revealed differences in the relative abundance (RA) of genes involved in the development and metabolism of *in vivo* and *in vitro* embryos [15], [16]. However, to our knowledge, no studies have exclusively compared the expression profile of genes specifically related to stress and apoptosis in bovine blastocysts, which are important parameters to consider in the assessment of embryo quality.

The analysis of transcripts from genes essential in early embryonic development provides a tool for the assessment of embryo quality and optimization of *in vitro* culture conditions and production protocols. Studies on *in vitro*-produced and cultured blastocysts exhibit alterations related to intercellular contact, compaction, differentiation, cell stress, and apoptosis. In addition, although mRNA expression patterns are highly conserved, there are crucial differences in the RA of transcripts of genes involved in blastocyst development, which may partly explain the differences in the quality of such embryos [13], [14], [17], [18].

The ability to identify precise changes in the expression profile of genes involved in both the response to cellular stress and the early stages of apoptosis would benefit *in vitro* embryo production by enabling, for example, the analysis of the physiological status of oocytes and embryos produced by different maturation and *in vitro* culture systems. The objective of the present study was to analyze the RA of a set of genes involved in cellular stress (heat shock protein 70-kDa, HSP70), endoplasmic reticulum (ER) stress (immunoglobulin heavy chain binding protein, Bip; proteasome subunit β5, PSMB5) and apoptosis (*BCL-2* associated X protein, Bax; cysteine aspartate protease-3, Caspase-3) in bovine blastocysts produced by IVF or SCNT and compare it with that of their *in vivo* counterparts.

**Figure 1:**
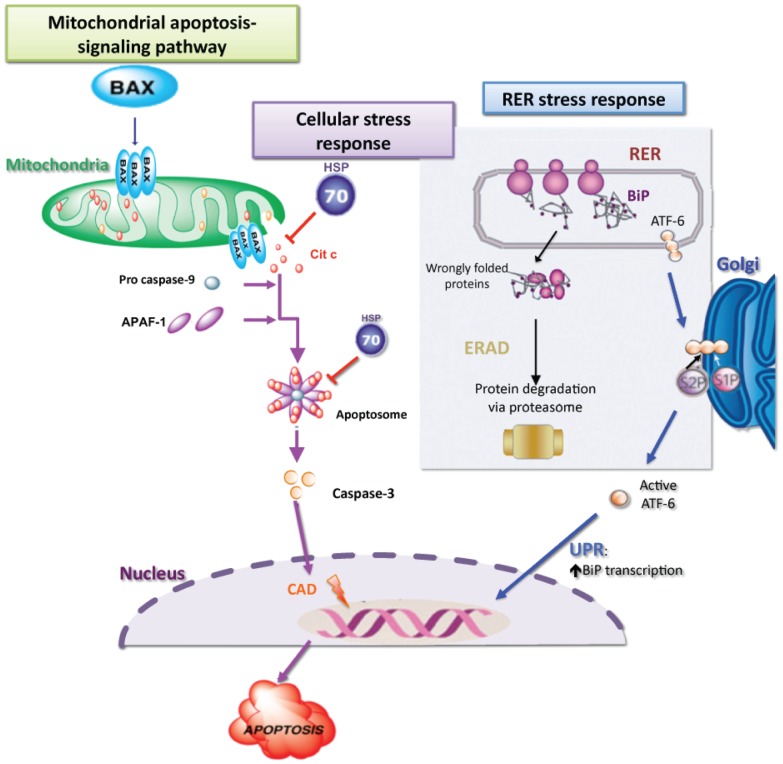
Signaling pathways active in cellular stress conditions.

Heat shock protein 70-kDa, HSP70, is a prominent cytoprotective factor that confers tolerance as a consequence of cellular stress or transfection. HSP70 expression inhibits the induction of apoptosis, thus conferring protection to damaged cells [19]–[21]. Therefore, monitoring the expression pattern of this gene contributes to understanding the physiological state of a cell or organism. Immunoglobulin heavy chain binding protein, Bip, is a chaperone member of the HSP family located in the lumen of the rough ER [22], and PSMB5 eliminates aberrant or misfolded proteins as a result of stress within the ER. Conditions inducing apoptosis [23]–[26] as well as gene expression analysis of apoptosis associated genes [27]–[28] are well studied in bovine preimplantation embryos. Cysteine aspartate protease-3, Caspase-3, is responsible for the activation of caspase-activated DNase (CADs) for DNA fragmentation [1] and Bax is a pro-apoptotic gene member of BCL-2 family genes. Both of these genes are involved in early stages of apoptosis, which can occur prior to any visible changes in morphology (Figure 1).

Since classical methods for gene transcript detection require large amounts of initial RNA, they are not suitable for application in oocytes and embryos [3], [29]. However, qRT-PCR is a highly sensitive technique that allows determining the RA of a target transcript as well as simultaneously amplifying an endogenous gene to be used as control.

The aim of the present work was to compare the RA of selected transcripts involved in cellular stress, ER stress, and apoptosis by qRT-PCR. This assay may be used to complement morphological analyses, ultimately providing a quantitative technique to assess the developmental potential and quality of bovine embryos produced by application of assisted reproductive technologies.

## Materials and Methods

### Chemicals, reagents and culture media for in vitro embryo production

All chemicals and reagents were purchased from Sigma Chemical Co. (St. Louis, MO, USA), unless otherwise indicated.

### Ethics statement

The protocol for this study (reference number 02/2011) was approved by the Committee on the Ethics of Animal Experiments of the Universidad Nacional de San Martin. Protocol development was approved as recommended by the National Institutes of Health (NIH) Guide for the Care and Use of Animals.

### Collection and in vitro maturation of bovine oocytes

Bovine ovaries from VISOM S.A slaughterhouse (Los Polvorines, Buenos Aires) were transported to the laboratory within 3 h at 25°C in sterile saline solution supplemented with 0.1 mg/mL streptomycin. For aspiration, follicles 2–10 mm in diameter were punctured using 19-G needles connected to a vacuum pump. Follicular fluid containing cumulus-oocyte complexes (COCs) were collected in sterile 50-mL centrifuge tubes (Corning, New York, USA). Only COCs with homogeneous cytoplasm and at least three layers of unexpanded cumulus cells were selected for *in vitro* maturation (IVM) [30]. Forty to fifty COCs were cultured in 400 µl of maturation medium and incubated at 38.5°C in 5% CO_2_ in air with maximum humidity for 24 h prior to their use. The maturation medium consisted of M199 supplemented with 100 µM cysteamine, 0.2 mM sodium pyruvate, 100 U/mL penicillin, 100 µg/mL gentamicin, 25.1 mM NaHCO_3_, 0.1 IU recombinant human follicle-stimulating hormone (FSH) (Puregón, Organon, Ca, USA) and 10% fetal calf serum (FCS) (Gibco, New Zealand).

### *In vitro* fertilization (IVF) procedure

Frozen–thawed semen from Brangus bulls with proven performance in IVF was used. All samples (500-µl straws) from each bull belonged to the same commercial batch. Thawing was performed by immersing straws for 30 s in a 37°C water bath.

Matured COCs were transferred to new four-well plates containing 400 µl of *in vitro* fertilization-synthetic oviductal fluid (IVF-SOF) medium, containing 107.7 mM NaCl, 7.1 mM KCl, 12 mM KH_2_PO_4_, 5 mM NaHCO_3_, penicillin 25 UI, 73 mM sodium pyruvate, 3% BSA, 2 mM fructose, 18 mM CaCl_2_2H_2_O, and 54 mM sodium lactate, supplemented with 50 µg/mL heparin. Motile sperm were separated using the Percoll gradient (90%/60%/30%) method. The thawed semen was centrifuged at 600 g for 20 min. Spermatozoa were washed twice using Hepes buffered-TALP, H-TALP (114 mM NaCl, 3.1 mM KCl, 0.4 mM NaH_2_PO_4_.H_2_O, 10 mM sodium lactate (60%), 25 mM NaHCO_3_, 1.4 mM caffeine, 2 mM CaCl_2_.2H_2_O, 0.5 mM MgCl_2_.6H_2_O and 10 mM HEPES) and centrifuged at 200 g for 5 min. Spermatozoa were resuspended in IVF-SOF medium. Finally, sperm cells were added to reach a final concentration of 2×10^6^ cells/mL and co-incubated with COCs for 24 h in a humidified atmosphere of 5% CO_2_ in air at 38.5°C.

### In vitro culture

Presumptive zygotes were denuded 24 h after fertilization by gently vortexing for 3 min. After removal of all of the adhering cumulus cells, presumptive zygotes were placed into four-well plates in groups of 50 in 400-µl drops of synthetic oviductal fluid (SOFaa) under mineral oil. Culture medium SOFaa consisted of 5 mM NaHCO_3_, 107.7 mM NaCl, 7.1 mM KCl, 1.2 mM KH_2_PO_4_, 1.5 mM MgSO_4_, 7.3 mM sodium pyruvate, 0.2 mM L-glutamine, 1.8 mM sodium citrate, 1.8 mM CaCl_2_2H_2_O, 5.4 mM sodium lactate, 1X essential aminoacids, 1X non-essential amino acids, 2.8 mM de myoinositol, 5 mg/mL BSA. Embryos were cultured for 7 days at 38.5°C in 5% CO_2_, 5% O_2_, 90% N_2_ with maximum humidity (Sanyo MCO 175M, Japan). Only morphologically normal embryos were included in the study.

### Somatic cell nuclear transfer (SCNT)

Primary bovine fetal fibroblasts (BFF) were established from a day 25 postconception fetus by disaggregation of whole body without head and viscera and cultured in DMEM (Gibco) supplemented with 10% fetal FCS at 38.5°C in 5% CO_2_ and humidified air. All procedures were performed as previously described [31], with minor modifications. After 18 h of maturation, loosely associated cumulus cells were removed by vortexing for 1–2 min in Hepes-buffered SOF (H-SOF) (SOFaa supplemented with 20 mM HEPES) containing 1 mg/mL hyaluronidase. Denuded oocytes were washed and returned to maturation medium. Enucleation process was initiated within 30 min of oocyte denudation under an inverted microscope (Nikon Eclipse TE-300, Nikon, Japan) and micromanipulators (NT 88 V3, Nikon Narishige, Japan).

Oocytes were incubated for 5–10 min in H-SOF supplemented with 1 mg/mL Hoechst 33342 at 38°C, subsequently placed into manipulation drops (H-SOF supplemented with 1% FCS covered with mineral oil) and enucleated after a brief exposure to UV light (Nikon Filter Set 01; Nikon, Tokyo, Japan) to determine the location of DNA.

Passage 3 donor BFF cells were collected from the culture plates by trypsinization using 0.05% trypsin-EDTA, washed twice, and finally resuspended in H-SOF. Cells were picked up with the transfer needle and slipped into the perivitelline space of enucleated oocytes. Cell-cytoplast couplets were fused immediately after cell transfer using a 0.5-mm gap fusion chamber (BTX, San Diego, CA, USA) overlaid with sorbitol fusion medium (0.25 M sorbitol, 0.5 mM magnesium acetate, 0.1% BSA) with 25 µsec of 2.4 kV/cm pulse (BTX Electrocell Manipulator 630, Harvard Apparatus, MA, USA). The post-fusion culture medium consisted of H-SOF supplemented with 5% FCS and 5 mg/mL cytochalasin B. After 1 h, any non-fused couplets underwent a second fusion procedure as described above. Twenty minutes after the second fusion procedure, fused couplets were activated by the exposure of the structures to 5 µM ionomycin in H-SOF medium for 4 min, then rinsed three times in H-SOF and allocated to a 4-h culture in 2 mM 6-DMAP at 38.5°C in 5% CO_2_ in air with maximum humidity. After this treatment, presumptive embryos were rinsed in H-SOF and cultured as described previously.

### *In vivo* production of embryos

Braford and Brangus cows from the experimental herds of the Instituto de Investigaciones Biotecnológicas (Buenos Aires, Argentina) were superovulated using an intravaginal device with 1 g medroxyprogesterone (Syntex, Argentina). On the same day (day 0), cows were administered with an IM dose of 30 IU of progesterone (Rio de Janeiro, Argentina) and 27 mg of estradiol benzoate (estradiol-17β) (Rio de Janeiro, Argentina). On day 4, cows received an ovarian stimulation protocol twice daily for four days (AM-PM, every 12 h), with decreasing concentrations of (FSH) (Folltropin, Bioniche, Canada) as follows: 40 mg on day 4, 30 mg on day 5, 20 mg on day 6, and 10 mg on day 7. On day 7, the intravaginal device was removed and cows were administered with 500 mg of coprostenol (a synthetic analog of prostaglandin F2a) (Syntex) and ovulation was induced with GnRH (Gonasyn, Syntex). On day 9, double inseminations were performed, separated by an interval of 8 h. The embryos were recovered on day 16 of the protocol (7 days after artificial insemination) using a uterine three-way lavage Foley catheter and 1 L of wash medium phosphate buffered saline (PBS), supplemented with 50 µg/mL kanamycin. At the end of embryo recovery, donors received 2 mL of coprostenol to avoid pregnancies of unrecovered embryos.

### Real-time reverse transcription polymerase chain reaction (qRT-PCR)

Groups of 10 embryos from the three sources (*in vivo*, IVF, SCNT) were homogenized in TRIzol reagent. To increase total RNA concentration, 3.5 µg of yeast tRNA was added as a carrier (Invitrogen, Life Technologies, Carlsbad, CA, USA) using a tissue homogenizer. Total RNA was prepared from TRIzol homogenates according to the manufacturer's instructions. Briefly, Poly(A)^+^ RNA was purified from total RNA using the PolyA Tract mRNA Isolation System (Promega, Madison, WI, USA) with the only modification being a 10-fold reduction in recommended reagent amounts for all steps [32]. Messenger RNA (mRNA) was transcribed using Superscript II enzyme (Invitrogen, Life Technologies) following the manufacturer's instructions. After reverse transcription, all the samples were diluted accordingly (1/2, 1/10) and used as template. The RT-qPCR reactions were carried out in an Applied Biosystems 7500 Real-Time PCR System (Applied Biosystems, Foster City, CA, USA). Primer sequences were designed using Primer Express Software v3.0 (Applied Biosystems Foster City, CA, USA). We selected the primer sets that amplified the sequences as close as possible to the 3′ end of the target genes (Table 1). Amplicons were 60–100 bp long. The reactions were carried out with the SYBRGREEN PCR Master Mix (Invitrogen) [32]. To verify that the SYBR Green dye detected only one PCR product, all the reactions were subjected to the heat dissociation protocol after the final cycle of the PCR [33]. All the samples were tested against Glyceraldehyde3-phosphate dehydrogenase (*GAPDH*) as a reference gene for data normalization. Each qRT-PCR quantitation experiment was performed in triplicate for three independently generated cDNA templates. Group means were analyzed for statistical significance using a t-test with the software Analyse-it for Microsoft Excel (Analyse-it Software, Ltd. England, UK).

**Table 1:** List of primers used for qRT-PCR for the specific selected genes.

Gene identification	Primer sequences (forward and reverse, 5→3)	Accession Number	Product length (bp)
*BAX* (*BCL-2* associated X protein)	F: GCGCATCGGAGATGAATTG	NM_173894.1	59
	R: CCACAGCTGCGATCATCCT		
*Caspase-3* (cysteine aspartate protease)	F: TACTTGGGAAGGTGTGAGAAAACTAA	NM_001077840.1	71
	R: AACCCGTCTCCCTTTATATTGCT		
*HSP70* (heat shock protein 70 - kDa)	F: CCATCTTTTGTCAGTTTCTTTTTGTAGTA	NM_203322.2	75
	R: GGAAGTAAACAGAAACGGGTGAA		
*BIP* (immunoglobulin heavy chain binding protein)	F: GATTGAAGTCACCTTTGAGATAGATGTG	NM_001075148.1	85
	R: GATCTTATTTTTGTTGCCTGTACCTTT		
*PSMB5* (proteasome subunit β5)	F: GCTTCTGGGAGAGGCTGTTG	NM_001037612.1	69
	R: CGGAGATGCGTTCCTTGTTT		
*GAPDH* (Glyceraldehyde3-phosphate dehydrogenase)	F: GGTTGTCTCCTGCGACTTCAA	NM_001034034.1	64
	R: AATGCCAGCCCCAGCAT		

### Statistical analysis

Statistical analyses were conducted using InfoStat Software for Windows (Universidad Nacional de Córdoba, Argentina). Treatment effects were assessed by one-way or two-way ANOVA followed by Tukey multiple-comparison post-hoc test to identify individual differences between means. When the data exhibited a skewed distribution, square-root transformations of the basic variables were performed before the statistical analysis. The experimental groups were always analyzed in parallel. The number of biological (n) material was established as previously described [13]. All values are presented as means with their corresponding SEM. Statistical significance was set at P≤0.05.

## Results

### *In vitro* embryo production

A total of 37 embryos were produced by IVF, with a global blastocyst production efficiency of 22.6%, whereas 30 embryos that were generated by SCNT, exhibited an overall efficiency of 18% (Table 2). Finally, 30 *in vivo*-derived embryos were used as control (Table 3).

**Table 2:** Global efficiency of assisted reproductive technologies on cleavage and blastocyst rates.

Reproductive technique	Total Oocytes	Total 2-cell embryos on day 2 (%)	Total Blastocysts/presumptive zygotes on day 8 (%)
IVF	196	164 (83.7) 160 (59.7)	37 (22.6) 30 (18.8)
SCNT	268		

a Data are expressed as mean ± standard error of the mean (SEM). B Three replicates were performed.

**Table 3:** *In vivo* embryo production.

Cow	Donor 1	Donor 2	Donor 3	Donor 4	Donor 5	Total
No. of blastocysts	8	6	7	4	5	30

Embryos produced through assisted reproductive technologies and *in vivo*-derived embryos were compared using differential staining. No statistical differences were found between the different sources of blastocysts with regard to cell numbers (data not shown).

### Gene expression analysis

**Figure 2:**
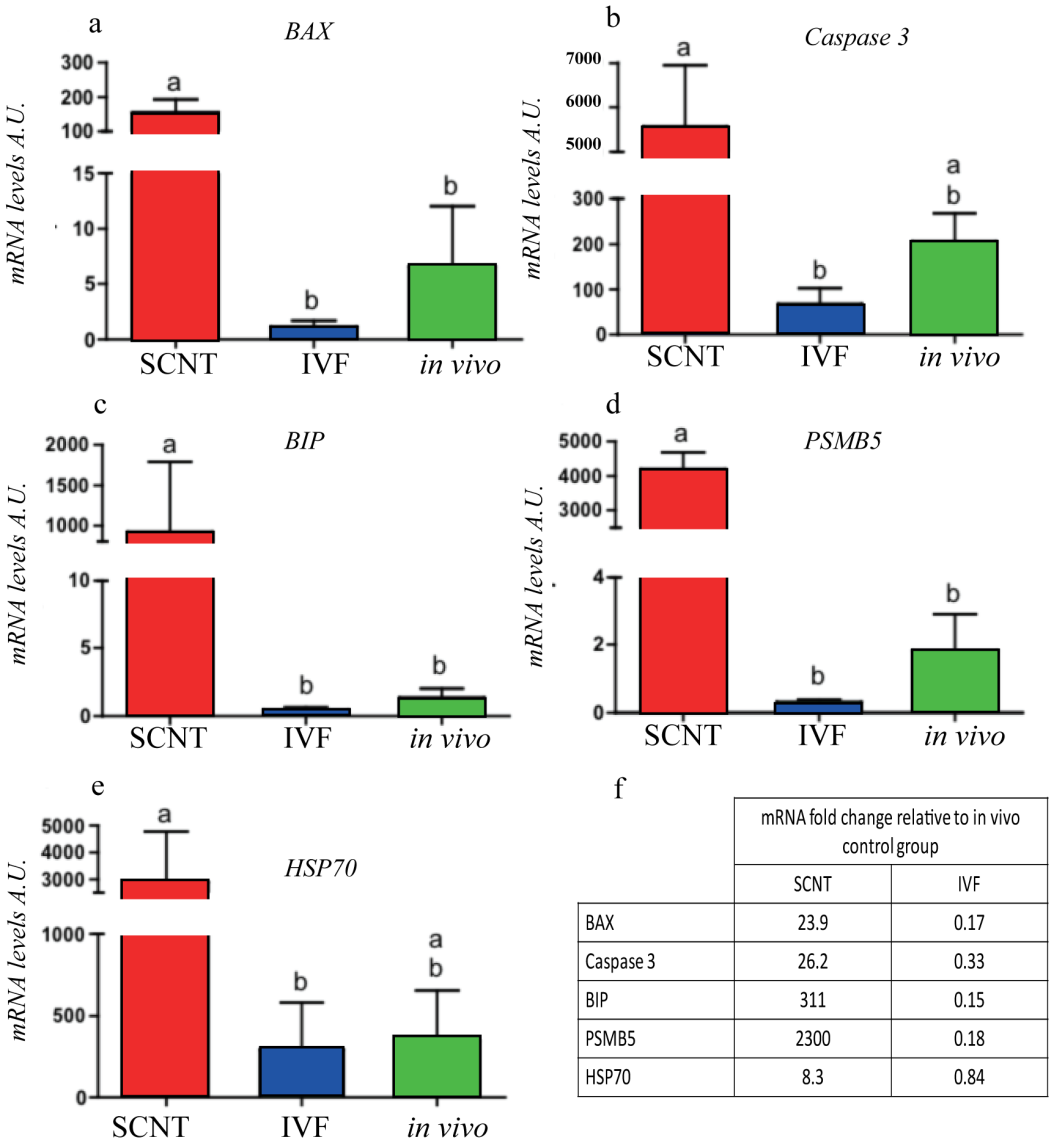
Quantification of mRNA levels for (a) Bax (b) Caspase-3 (c) Bip (d) PSMB5 proteasome subunit β5 and (e) HSP70 by real-time RT-PCR in bovine blastocysts produced either *in vitro* (IVF or SCNT) or *in vivo*. (f) Table of mRNA expression levels presented as fold change relative to control embryos. Normalized transcription levels are shown as mean ± standard error of the mean (SEM). Different superscripts indicate statistical differences between treatment groups (*P*<0.05). Data were obtained from three replicates of independent groups of 10 embryos each.

A series of real-time PCR assays was established to detect mRNA expression levels of Bax, Caspase-3, Bip, PSMB5, and HSP70. The RA of transcripts from SCNT- and IVF-derived embryos were compared with that of *in vivo*-produced control embryos. The expression levels of the pro-apoptotic Bax gene were significantly higher (p = 0.0001) in SCNT-derived embryos (23.9 fold higher) that the *in vivo* blastocyst group (Figure 1a). On the other hand, for IVF and *in vivo* control embryos, we found no statistical differences in Bax expression (Figure 2a). Similar mRNA expression profiles were observed in blastocysts from the three sources for the Bip and PSMB5 genes (Figure 2c, d). The RA of transcripts for PSMB5 was significantly increased (p<0.0001) in SCNT blastocysts compared with the *in vivo* produced group. In parallel, as illustrated in Figure 2c, the expression levels for Bip were 311 fold higher in SCNT embryos than in the control group (p = 0.0209). Although the standard deviation between the groups of blastocysts produced by SCNT was considerable, embryos produced either by IVF or *in vivo* generally showed significantly (p<0.05) lower RA of transcripts for Bax, Bip and PSMB5 than embryos produced by SCNT.

Interestingly, while higher gene expression was observed for the selected genes in SCNT embryos compare to the *in vivo* derived control embryos, the IVF embryos had a tendency for lower levels of expression of these genes compare to control. No statistical differences were found between the assisted reproductive technique IVF and *in vivo* control embryos with regard to the Caspase-3 and HSP70 gene expression patterns (Figure 2b,e). However, the RA of mRNA for these two genes in SCNT-derived embryos was higher than that of the IVF group, 26.2 vs 0.33 and 8.3 vs 0.84 for Caspase-3 and HSP70, respectively, as compared to the control group.

Our results led us to focus on the genes selected as possible markers for embryonic stress. As shown in Figure 2, the SCNT technique significantly enhanced the expression levels of the genes selected.

## Discussion

It has been shown that the use of several assisted reproductive technologies such as IVF and SCNT can affect embryo quality in terms of gene expression and epigenetic marks in oocytes and embryos as compared with *in vivo*-produced embryos [34], [35]. Large offspring syndrome (LOS) is a molecular phenomenon characterized by difficult parturition due to increased birth weight of the calves, fetal and neonatal losses, or an unhealthy status, occasionally reported in *in vitro*-produced embryos [30]. As indicators of embryo quality and viability, we analyzed a group of key genes involved in the response to generalized cellular stress, ER stress, and regulation and activation of programmed cell death. We compared the mRNA expression profiles for Bax, Caspase-3, Bip, PSMB5, and HSP70 in bovine embryos that were produced by different assisted reproductive technologies.

In recent years, temporal expression profiles of various gene transcripts have been studied at different stages of embryonic development [17], [35], [36], [37]. The genes analyzed in these previous studies are involved in the pre- and post-implantation processes of embryo development, as compaction and cavitation (E-cadherin, Connexin 41 and 43), metabolism (Glut 5, Glut 1, IGF II, G6PDH), DNA methylation (DNMT), oxidative stress (SOX, SOD and MnSOD), apoptosis (Bax, caspase-7 and p53), trophoblast integrity (INFτ) and signs of growth factors (IGF and IGFR). To ensure non-maternal origin, we analyzed the RA in embryos that were at the blastocyst stage, where the levels of mRNA and transcriptional regulator factors are under the control of embryonic genome. Our results show that the RA of three of the stress indicators studied was significantly increased in SCNT embryos as compared with that of *in vivo*-derived blastocysts for Bax, PSMB5 and Bip.

Previous reports have shown a correlation between the effect of the culture system on the incidence of apoptosis and the level of the pro-apoptotic gene, Bax, in the quality and clinical normality of *in vitro*-produced embryos [32]. The RA of Bax is significantly higher when SOF medium is used for cultures of *in vitro*-derived bovine blastocysts compared with the *in vivo*-produced counterparts. Blastocysts co-cultured in the presence of somatic cells also show up-regulated Bax levels [2], [13], [38]. Moreover, it is noteworthy that this gene is also overexpressed in *in vitro* bovine blastocysts cultured in SOF medium supplemented with serum [39].

Furthermore, it has been suggested that the interaction of the expression of Bax and HSP70 may occur under stress conditions, with suppression of Bax activation in cells with high HSP70 levels [40]. In the present study, we found no significant difference in the abundance of transcripts for HSP70 and Caspase-3. However, conflicting results have been reported and the potential use of mRNA expression of BCL-2, Bax, Caspase-3 and Caspase-7 as markers for apoptosis in bovine blastocysts has not been clarified in single embryo detection [41]. Previous reports have demonstrated that bovine blastocysts produced *in vitro* with low amounts of activated group II caspase activity have increased potential for development to the hatched blastocyst stage [1]. These reports also suggest that determination of caspase activity may be a useful tool for the selection of embryos that are to be transferred into recipients [1].

The increased expression of PSMB5 and Bip found in the present study is perhaps not surprising, suggesting that SCNT-derived embryos were under conditions of ER stress. When ER stress conditions persist, initiation of apoptotic processes is promoted. Furthermore, the significantly higher transcript levels of Bax could imply initial activation of apoptotic mechanisms, but the low levels of Caspase-3 reveals that the apoptotic caspase cascade signaling system was not yet activated. This could be explained because caspases are secreted as inactive procaspases, which are only active after further modification. Biologically active caspase-3 and -7 are executioner caspases only activated during the process of apoptosis by active precursor caspases-8 and -9. Taking this into account, mRNA levels of caspases can only reflect the amount of procaspases, but not of the level of biologically active caspases. Very little is known on the exact timing of the successive steps in the apoptotic pathways of early embryos. An apoptotic response is thought to depend on the balance between cell survival and cell death inducers [42]. Here, the hatching rate based on the total number of blastocysts was similar in embryos resulting from mature oocytes that were either fertilized or enucleated to perform SCNT.

With regard to IVF-derived embryos, no significant differences were detected in the RA of the genes investigated as compared with that of *in vivo*-produced blastocysts. Besides the fact that higher gene expression was observed for the selected genes in SCNT embryos compare to the *in vivo* derived control embryos, the IVF embryos had a tendency for lower levels of expression. This could be explained considering that genes expressed in the IVF embryos may be specifically affected by *in vitro* oocyte maturation and fertilization. Control embryos are produced from oocytes matured and fertilized *in vivo*. Previous studies compared in vivo matured, fertilized, developed (AI) and *in vivo* matured, fertilized but *in vitro* cultured (IVD) embryos to solely *in vitro* produced IVF embryos, they discovered that genes involved in RNA processing and binding were differentially expressed, that is, down-regulated in the IVF embryos [43]. Moreover, gene expression differences between the IVF and SCNT embryos may be partially attributed to developmental competence variability (compared with *in vivo* embryos), as well as the maternally inherited genetic variability among IVF embryos.

Differential mRNA expression in bovine blastocysts obtained by different reproductive techniques produces blastocysts of divergent quality. Even though the embryos were morphologically comparable, classified as Grade 1 (excellent quality), the mRNA expression pattern was dissimilar. The RA differences found may explain the lower developmental capacity of SCNT embryos following transfer, which adversely affects their ability to establish pregnancy and produce a live calf.

## Conclusions

The present work analyzes the RA of a set of genes involved in cellular stress (HSP70), ER stress (Bip, PSMB5), and apoptosis (Bax, Caspase-3) in bovine blastocysts produced by IVF or SCNT as compared with that of their *in vivo* counterparts. The results presented in this paper propose a new set of stress-indicator genes that provide a widespread idea of the quality of pre-implantation bovine embryos. The apoptosis incidence in embryos is influenced mainly by the conditions and characteristics of maturation of the oocyte from which they are originated [1].

In this study, the oocyte *in vitro* maturation, as well as the embryo *in vitro* culture processes for the IVF and SCNT embryo generation followed exactly the same procedures. This allowed alleviating the potential biases in the comparison of gene expression patterns due to IVC conditions. The high incidence of pregnancy loss and neonatal death after SCNT has been hypothesized to result from incomplete nuclear reprogramming. Several studies have compared gene expression in somatic donor cells and the embryos resulting from them [44], [45]. In the present study, we observed higher gene expression for the selected genes in SCNT embryos compare to the IVF, and the *in vivo* derived control embryos. We cannot rule out the possibility of incomplete nuclear reprogramming in our donor cells. In a previous study, a comparisson of global gene expression profiles of individual bovine SCNT blastocysts with their somatic donor cells and fertilized control embryos suggested that the commonly observed low developmental efficiency of NT embryos may not be largely due to nuclear reprogramming during early embryo development (reprogramming of the somatic donor cell genome from a differentiated to a totipotent status, i.e., gene dedifferentiation) but may be potentially caused by abnormal gene reprogramming during postimplantation fetal/placental development [45]. Overall, our study could potentially complement morphological qualitative analysis by helping to develop an efficient, effective, and accurate technique to diagnose embryo quality, ultimately aiding to improve the efficiency of artificial reproductive techniques.
